# Increasing Environmental Health Literacy through Contextual Learning in Communities at Risk

**DOI:** 10.3390/ijerph15102203

**Published:** 2018-10-09

**Authors:** Leona F. Davis, Mónica D. Ramirez-Andreotta, Jean E. T. McLain, Aminata Kilungo, Leif Abrell, Sanlyn Buxner

**Affiliations:** 1 Department of Teaching, Learning and Sociocultural Studies, University of Arizona, 1430 E. Second St, Tucson, AZ 85721, USA; leonafdavis@email.arizona.edu (L.F.D.); buxner@email.arizona.edu (S.B.); 2 Department of Soil, Water and Environmental Science, University of Arizona, 1177 E. Fourth St, Tucson, AZ 85721, USA; mclainj@email.arizona.edu (J.E.T.M.); abrell@email.arizona.edu (L.A.); 3 Water Resources Research Center, University of Arizona, 350 N. Campbell Ave, Tucson, AZ 85719, USA; 4 Mel and Enid Zuckerman College of Public Health, Health Promotion Sciences Department, University of Arizona, 1295 N. Martin Ave, Tucson, AZ 85721, USA; paminata@email.arizona.edu; 5 Department of Chemistry and Biochemistry, University of Arizona, Tucson, AZ 85721, USA

**Keywords:** environmental health literacy, environmental health risk, informal education, contextual learning, rainwater harvesting, program evaluation, hazardous waste sites, environmental justice communities

## Abstract

Environmental health literacy (EHL) has recently been defined as the continuum of environmental health knowledge and awareness, skills and self-efficacy, and community action. In this study, an interdisciplinary team of university scientists, partnering with local organizations, developed and facilitated EHL trainings with special focus on rainwater harvesting and water contamination, in four communities with known environmental health stressors in Arizona, USA. These participatory trainings incorporated participants’ prior environmental health risk knowledge and personal experiences to co-create training content. Mixed methods evaluation was conducted via pre-post participant surveys in all four trainings (*n* = 53). Participants who did not demonstrate baseline environmental science knowledge pre-training demonstrated significant knowledge increase post-training, and participants who demonstrated low self-efficacy (SE) pre-training demonstrated a significant increase in SE post-training. Participants overall demonstrated a significant increase in specific environmental health skills described post-training. The interdisciplinary facilitator-scientist team also reported multiple benefits, including learning local knowledge that informed further research, and building trust relationships with community members for future collaboration. We propose contextual EHL education as a valuable strategy for increasing EHL in environmental health risk communities, and for building academia-community partnerships for environmental health research and action.

## 1. Introduction

Communities that are disadvantaged by dominant social, economic, and political systems also often suffer disproportionately from environmental health risk [1,2,3]. Additionally, the increase of extreme and unpredictable weather events due to climate change is further widening this environmental health risk disparity [4,5,6]. Environmental justice (EJ) integrates the many potential layers of subjugation these communities face; for example, residents may suffer from discriminatory land use planning, limited access to health care, limited employment opportunities, and substandard sanitation infrastructure [2,7]. Though we acknowledge the four partnering communities in this study are EJ communities (Table 1), we use the term “environmental health risk communities” here to locate the specific study emphasis on environmental health, and to highlight environmental health risk as the focus of our partnership with these communities.

The natural interdependence of science literacy, health literacy, and environmental literacy have led to the evolution of current environmental health literacy (EHL) frameworks [8,9,10,11,12,13]. The Society for Public Health Education states that EHL “integrates concepts from both environmental literacy and health literacy to develop the wide range of skills and competencies that people need in order to seek out, comprehend, evaluate, and use environmental health information to make informed choices, reduce health risks, improve quality of life and protect the environment” [14]. As the mitigation or prevention of community-level environmental health risk often requires coordinated action, scholars have identified the need for EHL to not only include knowledge and efficacy for personal environmental health actions, but also for community level action [8,12,15]. Case studies have demonstrated increasing EHL as an effective strategy to equip environmental health risk community members to develop and implement their own contextually appropriate strategies for addressing environmental health risk [9,12,16,17,18]. Thus, increasing EHL in environmental health risk communities is a strategy for realizing environmental justice.

**Figure 1:**
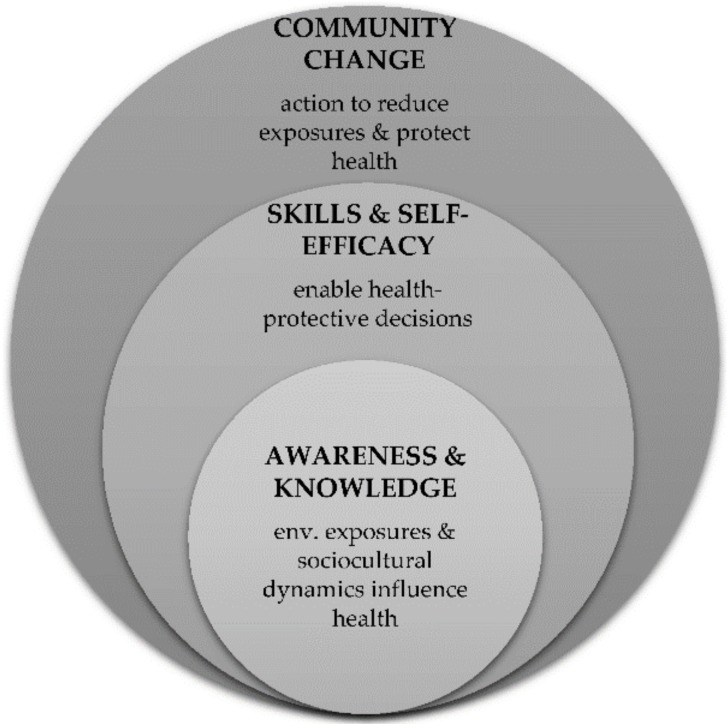
Three dimensions of environmental health literacy (EHL), as proposed by Gray [8].

Aligning with Gray’s (2018) proposed refinement to the definition of EHL [8], illustrated in Figure 1, we define EHL here as: (1) environmental science knowledge and awareness related to the community’s specific environmental risks; (2) skills and self-efficacy for learning science, doing science, and environmental action; and, (3) community action for systemic change.

Gray further suggests EHL as a “continuum” of learning that is often “tied to active engagement of participants throughout the research process, especially in communities directly impacted by environmental contamination” (p. 466). The contextual EHL trainings described and evaluated here represented both an educational event on their own, and a starting point for participants to continue learning alongside researchers through a citizen scientist program, if they chose to do so.

In a previous study, 74% of survey respondents reported that they get “some or a lot” of their science- and technology-related learning from “life experiences” [19]. The Contextual Model of Learning, as proposed by Falk and Dierking [20], asserts that, “learning can be conceptualized as a contextually driven effort to make meaning in order to survive and prosper within the world; an effort that is best viewed as a continuous, never-ending dialogue between the individual and his or her physical and sociocultural environment” [21]. Research has shown people learn more when content is grounded in local culture and context [22,23,24], and when participants’ knowledge, beliefs, and experiences are shared and honored in a participatory learning experience [16,17,25]. Previous EHL research has emphasized the contextual nature of defining and evaluating EHL, and the importance of matching learning content to specific local environmental health risk factors [17,26,27].

## 2. Materials and Methods

### 2.1. Contextual Training Design

The contextual EHL trainings discussed here were designed and promoted to residents of the four partnering communities as “Step 1” of participation in an environmental citizen science project titled Project Harvest. Project Harvest is a co-created citizen science project [28] aimed at assessing environmental contamination in harvested rainwater with environmental health risk communities, presently ongoing. The trainings discussed here served multiple purposes: (1) engage and educate environmental health risk community members around environmental health issues, and increase EHL in these communities, (2) provide an easy entry point for community members to volunteer as citizen scientists in Project Harvest, continuing to learn and contribute to research through local environmental monitoring, and (3) pilot the sampling methods, instructional materials, and survey methods, as formative evaluation for future Project Harvest participant materials.

In the initial project design phase of Project Harvest, principal investigators drafted the content for public community trainings to include climate change, energy conservation, air and water quality, microbiology of food and water, basic inorganic and organic chemistry, practical household actions for environmental health and environmental sustainability, and hands-on experiences that are related to collecting soil, water, and plant samples for environmental monitoring. These topics were chosen based on specific contamination issues in the partner communities, expressed concerns from community members through prior partnerships, and their importance to comprehension of relevant environmental health information.

### 2.2. Partner Community Selection

**Figure 2:**
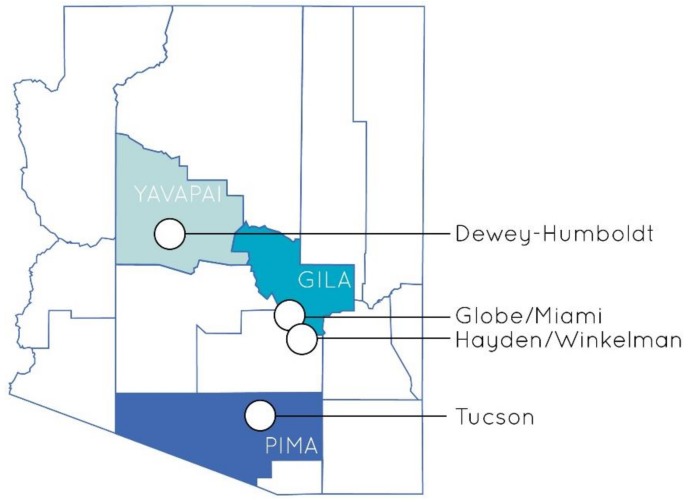
Partnering community locations in Arizona, USA.

Four communities, located in Arizona, USA, were selected to host these trainings based on their proximity to a federal Superfund site and other toxic release site(s), expressed interest from community members or local organizations, and previously built relationships between researchers and community members. The term “community” is used here to define a group with shared local source(s) of environmental health risk, though each defined community varies in size and municipal designation. For two of these communities, Hayden/Winkelman and Globe/Miami, two adjacent municipalities were considered as one community as they share common sources of environmental health risk and associated environmental health concerns, as well as similar demographics. Although the Superfund site located in Tucson, which is a significantly larger city, poses greater health risk to residents in closer proximity, the Tucson training was not restricted from any local residents for ethical reasons, and thus the City of Tucson is defined as a community here. Table 1 describes these four communities and rationale for their partnership for this study, and Figure 2 shows geographic locations.

### 2.3. Participant Recruitment

Recruitment for training participants was conducted primarily via: (1) information tables at community festivals, community group meetings, and/or town Council meetings; (2) follow-up mailings, telephone calls and emails to community members and local organizations; (3) press releases for local media outlets; (4) announcements in newsletters; and (5) the Project Harvest website. In every community, principal investigators participated in multiple events involving in-person engagement with community members. All promotional materials were produced in Spanish and English, to welcome and encourage Spanish-only speakers in addition to English speakers. See Supplemental Materials for examples of promotional flyers distributed. Table 2 details specific engagement methods that were conducted in each community.

### 2.4. Training Facilitation

Principal investigators of Project Harvest, representing research fields of Soil and Water Science, Microbiology, Organic Chemistry, Inorganic Chemistry, and Public Health, served as training facilitators, along with other specialists on climate change and rainwater harvesting. The term “facilitator-scientist” is used henceforth to refer to individuals in this role.

Community trainings were hosted in locally familiar public sites and scheduled either over 1–2 consecutive full days or over 3–5 consecutive mornings. See Supplemental Materials for the full agenda of each training. Tucson and Globe/Miami trainings were facilitated bilingually in English and Spanish, to accommodate Spanish-only speakers present. All of the participants in Dewey-Humboldt and Hayden/Winkelman trainings were English speakers. Four of the facilitator-scientists are Spanish-English bilingual and conducted their presentations and discussions in two languages. All other content was provided bilingually through:

1. Simultaneous translation during the lectures, with bilingual facilitator-scientists acting as translators.
2. Dual projection of slide presentations in English and Spanish (all slides were identical in content and presented and projected in English and Spanish).
3. Spanish language kit and manual during sampling hands-on education, and bilingual educators on site to provide one-on-one assistance.
4. English and Spanish take-away copies of all slides and supplemental education materials provided in a binder [43] (Supplemental Materials).

### 2.5. Research Methods

A 17-item survey consisting of multiple-choice and short answer questions and a 33-item Likert scale survey (see Supplemental Materials) were administered to all willing participants at the beginning and end of the training, to assess changes in participant EHL. Additionally, a pre-training survey to collect demographic information, and post-training survey to gain feedback on participants’ experience of the training, suggestions for future trainings, and current rainwater harvesting practice, were also administered. Participant feedback (both via survey and verbal) informed adjustments in training design and facilitation as the series of trainings progressed.

Of 67 total participants in the four trainings, 53 participants attended the entire training, completed both pre- and post-surveys, and consented under the University of Arizona IRB rule as an approved project. Of these, 14 participants were in Tucson, 15 were in Hayden/Winkelman, 14 were in Globe/Miami, and 10 were in Dewey-Humboldt. De-identified survey data included closed-ended question (Likert scale and multiple choice) responses that were recorded and analyzed quantitatively by one researcher and research assistant. Open-ended responses were reviewed and analyzed using validated qualitative methods by two research assistants and a supervising researcher. A coding scheme was created for each question that captured main concepts reflected in participant responses. The group met periodically throughout the coding process to revisit codebooks, sometimes choosing to create or merge categories based on emerging themes in the data. All survey data was organized in Microsoft Excel, and statistical analyses were performed while using Microsoft Excel and Statistical Package for Social Sciences (SPSS) software [44].

Table 3 outlines the specific data collected as it relates to each research question identified. The numerated list following Table 3 details specific analysis methods for each category of assessment.

### 2.6. Data Analysis Methods

1. Motivation to learn was measured through four questions on the pre-program participant survey asking participants why they chose to attend, what they hoped to gain from the program, about their current rainwater harvesting practices, and what prior training on similar topics they may have received. Participant responses were coded for common themes, and occurrence of themes were aggregated to understand common motivations for choosing to attend the training.
2. Attitude towards the environment was measured by two closed-ended survey questions about preference towards environmental protection and environmental investment. Participant responses were aggregated and evaluated as a group, and pre-post change was evaluated per participant.
3. Environmental science knowledge was measured through seven open-ended and six closed-ended survey questions. Responses to four multiple choice questions were scored for level of knowledge. Responses to seven short answer questions were analyzed for both specific knowledge concepts, using qualitative coding for themes; and, for level of knowledge, by assigning a 0 (no knowledge), 1 (partial knowledge), 2 (baseline knowledge), or 3 (advanced knowledge) to each response. Coding rules for level of knowledge were specific to the question being analyzed. Table 4 describes the coding rules and example responses to one open-ended survey question. A dependent samples *t*-test was performed per question to assess significant change in participants’ mean level of environmental science knowledge for each topic area. Because participants who scored below baseline knowledge pre-training had more opportunity for learning gains, an additional dependent samples *t*-test was performed to look at knowledge change in those participants only. For this test, questions that could only be scored right or wrong were omitted.
4. Skills for environmental health comprised a significant portion of training content. Three survey questions asked participants about specific actions they could take to (1) “curb the effects of climate change”, (2) “make a positive impact on water reliability in the future”, and (3) “protect the environment, conserve water, conserve energy, and protect the health of your family and neighbors”. Responses to these questions were coded for categories of environmental health action. The number of participants describing each skill category was compared pre- and post-training. Additionally, specific strategies described per participant pre- and post-training were averaged per participant and compared using a dependent samples *t*-test.
5. Motivation for environmental action was assessed by eleven Likert scale items. These items were modified from literature provided by the Cornell Lab of Ornithology [45], who use a similar measurement tool with their citizen science program participants. Five items were designed to measure external motivation for environmental action, and six items were designed to measure internal motivation. Following the recommended analysis methodology from Cornell, the sum of external motivation scores were subtracted from the sum of internal motivation item scores, to calculate an overall motivation score per participant. Positive scores indicate predominantly internal motivations, while negative scores indicate predominantly external motivations. A dependent samples *t*-test was used to analyze pre-post change in motivation for environmental action overall. A Wilcoxon Signed-Rank test was used to test for significance between the mean pre- and mean post-survey scores by community.
6. Self-efficacy (SE) is defined as a person’s belief in their own capabilities [46]. SE was measured using a Likert scale in the categories of “SE for learning science” (six items), “SE for doing science” (four items), and “SE for environmental action” (12 items). These items were modified from the literature provided by Cornell Lab of Ornithology [45], and analyzed using their suggested methodology. Four items on the survey were worded in reverse (statements reflected lack of SE), were therefore scored in reverse. From a total dataset of 41 paired surveys, 12 “high self-efficacy” participants were removed who met the criteria of having 15 or more responses of a 4 or 5 on the 1–5 Likert scale, to isolate the participants with an opportunity for change. Using this “low pre SE” dataset (*n* = 29), the mean SE for each SE category was calculated pre- and post-training for each participant, and were analyzed using the Wilcoxon Signed Rank test to assess differences between communities.
7. Community change is defined broadly here to include relationship building, network building, collaboration, educational advocacy, and political advocacy. Participant responses to three survey questions and reflections from facilitator-scientists were analyzed for community action themes. Additionally, the individual decision by a participant to further invest their time and effort as an environmental citizen scientist post-training was considered to be a community-level action, as participation in co-created citizen science involves translating results into responsive action as part of the study design [28].
8. Participant experience was assessed through qualitative coding for themes in responses to four open-ended survey questions related to participant perception of benefits of the training, their intentions to harvest rainwater, and suggestions for training improvements and future trainings.
9. Facilitator-scientist experience was assessed through an open-ended survey via email with four of the facilitator-scientists, all who were involved in the design and agenda planning for the trainings. Survey questions asked for general reflections, specific surprises or challenges in the trainings, and for specific anecdotes of participants connecting content to local knowledge or life experiences.

## 3. Results

Summary: The majority of training participants came to the training with intrinsic motivation to learn, high internal motivation for environmental action, and pro-environmental attitudes. Before and after the training, the majority of participants demonstrated baseline knowledge or above in the topics of greenhouse gases, climate change, scientific method, soil and water contamination, and contaminant transport. Participants who demonstrated below-baseline knowledge pre-training in the topics of climate change impacts, fossil fuel impacts, soil and water contamination, and chemical concentrations demonstrated significant knowledge gains post-training. Participants who demonstrated low self-efficacy (SE) pre-training demonstrated significant increase in all SE categories post-training. Survey responses reflecting community-level action for environmental health were present but not frequent. Participants’ reflections on the training experience emphasized gaining knowledge, strategies for environmental health, and greater appreciation for the environment and science. Facilitator-scientists reflected on the benefits of gaining first-hand contextual knowledge of an area from community members, building trust relationships with community members, and gaining a deeper sense of meaning in their work through learning directly with members of environmental health risk communities. The following subsections provide further detail.

### 3.1. Participant Demographics

Overall, training participants (*n* = 53) were 64% female, 36% male. 62% identified as White/Caucasian, 36% identified as Latino/Hispanic, with other ethnicities being represented by 6% or less of the group. Three participants in Tucson and one in Globe/Miami were Spanish-dominant speakers, the rest were English-dominant speakers. All four Spanish-dominant speakers cited English as a secondary language, and 14 English-dominant speakers cited Spanish as a secondary language. 60% of participants reported having a college degree, though the level of education ranged to high school only (10%). Using 2015 HUD Income Guidelines, 28% of participants reported living in households designated as “Low Income” or below ($47,200 annual gross income or less for a family of four). 76% of participants were aged 45 years or older and 26% of the total group were 65 years or older.

### 3.2. Participant Motivation and Attitude

Overall, participants who volunteered to attend these trainings showed a high level of motivation to learn, high internal motivation for environmental action, and pro-environmental attitudes before and after the training. In Hayden/Winkelman, 19 out of 22 participants were teachers at the local school and were attending as part of their professional role with endorsement from the school superintendent. In Tucson, four of participants are employed as *promotoras* (community health workers) with SERI, participating as part of their paid employment, as required training for their upcoming role in Project Harvest. Otherwise, participants had elected to attend on their own time. 54% of responding participants reported attending previous workshops related to the environment, water, or energy. Participants’ household rainwater harvesting or food gardening practices were often cited as reasons for wanting to better understand environmental health risks (48%). The desire to help the environment (34%), and to learn practical skills to improve environmental and personal health (22%) were also cited as reasons for attending. Some responses cited local knowledge or personal experience that motivated their interest in environmental health. As one Globe-Miami participant stated:

“*We dug a cistern and have harvested rainwater for 30 years. We live in the canyon where Dioxin/Agent Orange was sprayed in the Pinal [Mountain]s 1965–1969. Many problems associated with that. Interested in present contamination, also because we grow our own vegetables.*”

Participants indicated their motivation for environmental action via eleven Likert scale statements on a 1–5 scale. Pre-training, the mean internal motivation was 4.4, and mean external motivation was 3.1. Post-training, the mean internal motivation score did not change, and mean external motivation was 3.0. A dependent *t*-test using the mean internal-external motivation scores resulted in no significant difference pre-post (*p* > 0.05). Wilcoxon Signed-Rank test results demonstrated no significant pre-post change in motivation by community (*p* > 0.05).

Additionally, two survey questions asked for participants’ preference of environmental protection and environmentally sustainable investment. 84% of responses (*n* = 38) favored all environmental proposals in both the pre- and post-surveys. Only one participant had pre- and post-responses for both questions that consistently reflected negative attitudes towards environmental protection.

### 3.3. Environmental Science Knowledge

**Figure 3:**
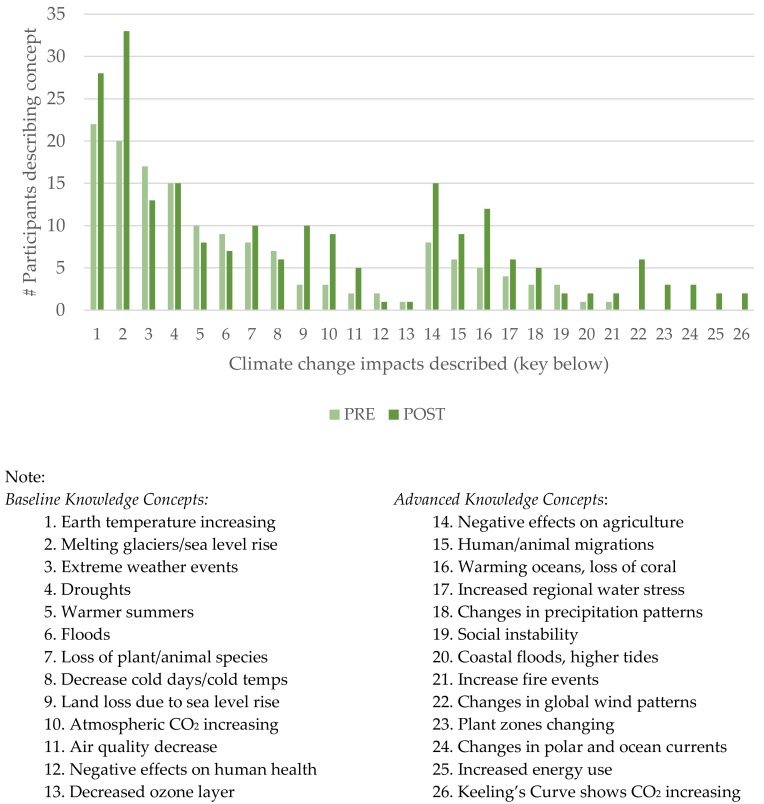
Described knowledge concepts of climate change impacts.

As outlined in Table 5 below, participants generally came to the training with at least baseline knowledge of greenhouse gases, climate change, scientific method, soil and water contamination, and contaminant transport. Significant knowledge gains were demonstrated in the topics areas of climate change, including increased specific concepts cited related to climate change causes and impacts (see Figure 3); and, in understanding chemical concentration nomenclature, which is critical to understanding environmental contamination information.

Isolating only participants who scored below the baseline level of knowledge on specific questions, significant knowledge increase was observed post-training related to impacts of climate change and fossil fuels, soil and water contamination, and chemical concentrations, as shown in Table 6.

**Figure 4:**
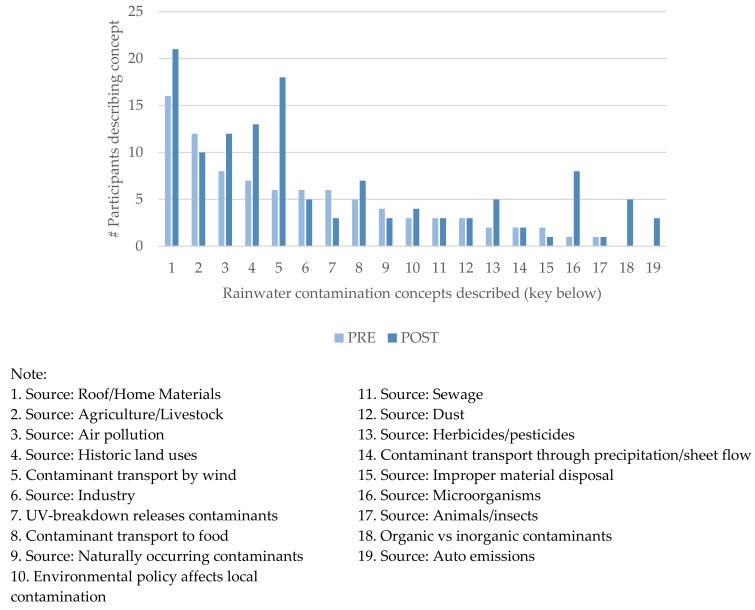
Described knowledge concepts related to rainwater contamination.

Although the level of knowledge related to soil and water contamination did not change significantly, the following environmental contamination concepts were described more frequently post- training, as illustrated in Figure 4: historic land uses as a contamination source, wind transport of contaminants, herbicides or pesticides as contaminants, microorganisms as contaminants, correctly naming organic (carbon-based) and inorganic (salts and metals) contaminants, and auto emissions as contaminants.

### 3.4. Self-Efficacy

Significant self-efficacy (SE) gains post-training were observed in all SE categories for participants who did not demonstrate high self-efficacy pre-training, with the largest gain in SE for doing science. This may be due to the hands-on portions of the training, where participants practiced sampling and analysis of water for contaminants, using laboratory equipment and following methods developed by professionals. Table 7 illustrates results of a Wilcoxon Signed-Rank test to assess pre-post change in SE by category.

### 3.5. Skills for Environmental Health Action

Analyzing paired surveys (*n* = 36), the mean number of specific skills mentioned per participant pre-training was 9.6, the mean skills mentioned per participant post-training was 13.2. A dependent samples *t*-test produced a significant (*p* < 0.01) increase post-training.

**Figure 5:**
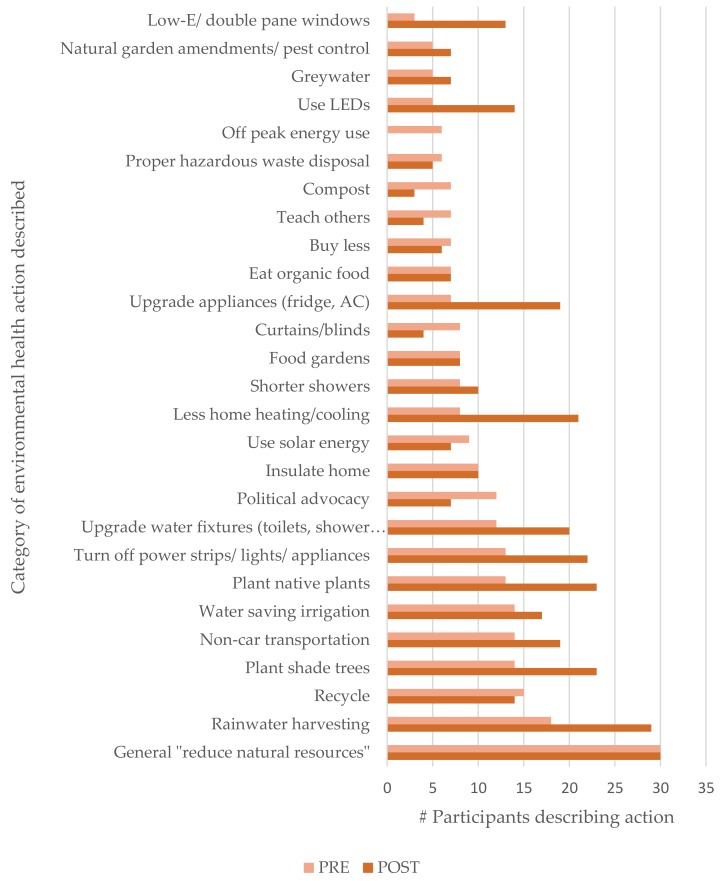
Most frequently described skills for environmental action.

General strategies, such as “conserve water” or “save energy”, were not counted as a “skill” towards the total skills cited per participant described above, though these general statements were summed under the category “General”. More participants described skills that were emphasized in training content in post-training responses, including rainwater harvesting, planting native plants and trees for shade, and home energy conservation practices. Figure 5 illustrates the most frequently described skills for environmental action pre- and post-training.

### 3.6. Community Change

The act of bringing interested people together around learning from each other is, at its core, a community action. The hands-on portions of the training allowed participants to build relationships and shared skills, and discussion portions allowed for participants to share personal and family experiences related to environmental health. One facilitator-scientist observed participants most often responding with stories or questions from personal experience following presentation content about specific contaminants of concern for the area.

As one facilitator-scientist reflected, “*During [the Globe/Miami] training, we would talk about the potential for heavy metal contamination of water—and the community members, who had lived under these huge smokestacks their whole lives, would tell stories of living and working in an environment where the mines were providing a living but were also potentially spewing toxins—which could get onto the roofs and wash into the rain barrels*”.

Another facilitator-scientist reflected, “*[In the Hayden/Winkelman training] we spent 20–30 minutes talking about their experiences as a child growing up in the area, swimming in the river with what they suspected to be tailings and waste water from the mine, their child’s lead biomonitoring data, and air quality*”.

The skills for environmental health that were described in Section 3.5 included two skills categories that emerged related to community action: social/political advocacy around environmental health issues, and teaching others about environmental health, as detailed in Table 8. Comments related to community action were mentioned by less than 5% of participants, though they were also not emphasized in training content nor being directly solicited by survey questions. However, this may also reflect a conceptualization of environmental health action limited to the individual or family scale for the majority of participants.

Six of the training participants had been already been hired as *promotoras*, or community health workers with citizen scientists in Project Harvest prior to the EHL training. One especially enthusiastic participant in Dewey-Humboldt was later hired as an additional community health worker for her community after the training. Nine other participants from the EHL trainings volunteered after the training for the three-year commitment of serving as a citizen scientist in Project Harvest, continuing to monitor local environmental contamination alongside other community members and university researchers.

### 3.7. Reflections from Participants

When asked, “What did you gain this week?” participants most often cited gaining scientific or environmental knowledge (69%), practical environmental health strategies or skills (44%), or greater awareness or appreciation for the environment and/or environmental science (40%). Examples included:

“*An appreciation of how things are connected. How these connections can be identified & monitored for protecting our public health*”—Globe/Miami participant

“*Calentamiento global, el peligro de algunos microorganismos. Importancia de conservar*” (Global warming, the risk of some microoganisms. Importance of conservation.)—Tucson participant

“*Deeper understanding of how and why CC [climate change] is occurring, stronger appreciation for the efforts of the scientific community, understanding and appreciation for the idea of “democratized science*”—Hayden/Winkelman participant

### 3.8. Reflections from Facilitator-Scientists

Reflections from a subset of facilitator-scientists (*n* = 4) largely reflected benefits that were gained from conducting in-person training with community members, categorized below.

#### 3.8.1. Formative Evaluation of Materials

Instructional booklets and sampling kits used in the trainings were piloted in part for their intended use with citizen scientists in Project Harvest the following year. Verbal and survey feedback from participants provided formative evaluation of these materials, and many suggestions were incorporated in material revisions or prompted the creation of new materials, such as video tutorials on sampling methodology.

#### 3.8.2. Gaining Local Knowledge to Inform Research

Spending time with community members in person allowed for scientists to gain understanding about community culture, beliefs, concerns, events, and history that informed the design of future research with these communities.

One facilitator-scientist described learning local contaminant history that researchers were not previously aware of: “*A husband and wife [in Globe/Miami] told us about how they suspected Agent Orange was sprayed in their neighborhood; we actually looked into this and confirmed indeed they had sprayed Agent Orange- there are a number of documents about it available* [47,48]. *So we added it to our contaminant list for analysis. There is a legacy of spraying by the federal government on Forest Service land southwest of Globe and on the nearby Apache Reservation east of town*”.

Another facilitator-scientist described learning of a concern community members had about local contamination study: “*A participant at one of the trainings shared a story where they had recruited a few fire fighters to join and do the training. They were excited and planned on attending, but were talked out of it by a firefighter’s wife. The wife stated that if the study observed any pollutants/elevated concentrations it would affect their ability to sell their home. The participant expressed frustration about their community and stated ‘…so when she said that I said, so your home value and selling your home is more important than your children’s health?’*” This anecdote highlights specific fears and social pressures present within the community.

#### 3.8.3. Building Relationship and Co-Creating Hope with Community Members

Relationships are built face-to-face, through dialogue and personal sharing. In rural areas especially, community members may feel distrustful of the motives and interests of research institutions. One facilitator-scientist reported some participants expressing skepticism about research motives being expressed in the trainings. However, in a face-to-face setting, she could explain her personal motivation for studying environmental health, and gain trust of community members. These personal relationships are immensely important for continued research in these communities with high environmental risk. Facilitator-scientists also expressed gaining a deeper sense of meaning in their work from the training experience. One stated, “*I derived a sense of hope by witnessing the participants engaged in learning our presentation topics”.* Another shared, “*To be perfectly honest, I approached this project as a scientist—in the beginning, I was much more interested in the water quality results, and the trainings/teaching was kind of secondary. But halfway through the first training I found myself really enjoying it—and ended up looking forward to the following trainings very much*”.

## 4. Discussion

### 4.1. Study and Survey Design

The mixed methods evaluation approach of using both closed-ended and open-ended survey questions, in addition to soliciting open-ended reflections from participants and facilitator-scientists, provided a comprehensive understanding of the training experience and outcomes from multiple perspectives. Additionally, facilitating trainings sequentially in four different communities provided valuable process feedback that allowed for continuous improvements.

Post-training surveys were administered immediately following the training experience, which limits our understanding of how the training might have contributed to participant knowledge, skills, and actions on a longer term. Additionally, all data was self-reported, which introduces the possibility for participant bias [49]. For example, participants may have felt social pressure to align with pro-environmental norms present in the training content.

Results reveal some limitations with the survey instruments used and inform recommendations for future survey design. The thirteen survey questions testing environmental science knowledge were specifically designed to vary in style and difficulty. However, closed-ended questions 9 and 10 (see Supplemental Materials) provided little value as almost all participants answered correctly both pre- and post-training, suggesting that the results might be due to “easy” question design rather than participant knowledge. Open-ended questions allowed for greater insight into participants’ thinking, though were more difficult to assign a quantitative level of knowledge to. For future assessments of environmental health knowledge, experience from this study informs the recommendation that survey design (1) Be as specific as possible to the local environmental health risks and contaminants in the community, and (2) Use matched open-ended and closed-ended questions that assess the same topic for the effective analysis of both level of knowledge and specific knowledge concepts and misconceptions.

Adhering to Bandura’s statement that there is “no all-purpose measure of perceived self-efficacy” [50] (p. 307), it proved successful to tailor previously tested Likert scales for SE [45] to the specific types of SE relevant to the training (learning science, doing science, and environmental action; all related to soil and water quality). The survey also included modified Likert scale statements for motivation for environmental action from the literature [45]. Grounded in self-determination theory [51], these motivation statements include separate items for internal and external motivations. We followed suggested analysis methodology from the authors of the original scale by subtracting external from internal motivation to achieve a “true” motivation score [45,52]. However, averaging all motivation items (internal and external) per participant produced similar pre-post change results. Acknowledging the sociocultural emphasis in contextual learning, and the value of peer-to-peer negotiation of knowledge observed in these trainings, we inquire whether external motivation might have real value in its influence on environmental action. This inquiry draws from recent research in science education, which places greater emphasis on discourse, social identity, and sociocultural context as primary, rather than background, elements of learning [53,54,55]. Furthermore, when community action may be necessary to address the specific environmental health risk posed, social motivation might play a valuable role in collective action, especially in rural areas [56]. Thus, measuring motivation for environmental health action may necessitate weighting external and internal motivations equally.

Questions assessing participants’ motivation to learn were open-ended and not designed for discriminating between internal and external motivation in responses. However, general motivation to learn and a pro-environmental attitude was observed in nearly all training participants pre- and post-training, suggesting that these qualities may be prerequisites for any volunteer-based EHL program.

### 4.2. Contextual Learning and Collaborative Relationships

In the recruitment, planning, facilitation, and evaluation of these community-based EHL trainings, the importance of contextual learning and teaching stands out. In each of these four communities, the unique history and culture dictated the appropriate manner of communicating environmental health information. In Globe/Miami, for example, the copper mine is both the main source of environmental health risk and the largest local employer. If a facilitator did not present environmental health risk information with knowledge and sensitivity to residents’ loyalty to the mine, then they would risk participants rejecting the information completely, and create barriers to future collaboration. However, by honoring local values and beliefs in the presentation of information, participants remain open to learning, even when information may challenge their perceptions. As most of the facilitator-scientists had limited prior experience with the partnering community, introducing a topic with guided open-ended questions (“How could your rainwater be contaminated?”) allowed for participants to provide culturally-appropriate content, often in the form of personal stories, that informed understanding of cultural context and allowed for connecting training content to participant experience and perceptions. At the conclusion of some training sections, the facilitator-scientist would pose the questions, “What else should we be measuring for? Is there anything else we need to know?” which allowed for participants to fill in gaps of the formal presentation with local knowledge. The critical role of local context in EHL education aligns with complementary research in the fields of health communication, environmental communication, and science education [17,23,25,27,57,58,59].

Environmental health risk communities might approach university researchers with distrust, as we experienced in this study. In all four communities, prior relationship and collaboration with reputable local organizations paved the way for further trust building with community members. In two communities, specific local individuals that were enthusiastic about the training program served as local “champions” [34] to motivate others. Additionally, a supportive learning environment, as observed in these trainings and prior research [60], is a critical element in a learner’s willingness to explore and accept new ideas and experiences. The importance of details in curating this kind of environment, from a familiar setting to supportive micro-interactions between community members and “experts”, cannot be understated.

### 4.3. Environmental Health Literacy and Community Action

Evaluating these trainings using the three dimensions of EHL, as proposed by Gray [8], described in the Introduction and outlined below, was instrumental in defining participant “learning” in the context of an environmental health risk community, as well as identifying opportunities and challenges in future EHL research.

#### 4.3.1. Knowledge and Awareness

As other scholars have discussed, EHL is highly contextual and it thus presents challenges for creating generalizable education or evaluation materials [17,26,27]. Relevant knowledge and awareness for EHL is specific to the community, the environmental health risks present, and the broader ecological, economic, and political dynamics of these risks. Although this reality poses challenges for defining and evaluating knowledge, it also forces us to critically examine what constitutes “relevant knowledge” for a given environmental health risk community. Whose knowledge? For whose benefit? In some cases, academic environmental health knowledge has been positioned in conflict with local knowledge, albeit unintentionally [61]. The face-to-face interactions between participants and facilitator-scientists in these EHL trainings were critical in facilitating the exchange of formal and informal knowledge as equal contributions to the construction of group knowledge.

To further honor the concept of knowledge as it is relevant to the community context, future survey evaluation of knowledge for EHL might benefit from moving away from formal science questions, towards more open-ended inquiry that allows for the community member to determine what knowledge is valuable. If a follow-up study with participants of these trainings was conducted, a question like, “What thoughts, ideas, or questions have you had since the training related to your health and environment?” may provide the most pertinent data.

Many previous environmental health interventions have disseminated health information from an authority (via brochures, website, etc.) as the primary strategy [62,63]. It is critically important that EHL education not approach community members as blank slates. Rather, new knowledge is mediated by prior beliefs and experiences [19,21], as well as through interaction within a social group [64,65,66]. Finn and O’Fallon describe EHL as “a process that individuals and communities embrace as a means of critical reflection within their local socioeconomic context rather than as a type of health literacy that incorporates specialized knowledge of environmental factors [10]”. Thus, what defines knowledge for EHL in an environmental health risk community should ultimately prioritize community concerns over academic biases, as has been skillfully demonstrated in some prior community-based environmental health research [16,25,67,68].

#### 4.3.2. Skills and Self-Efficacy (SE)

As learning leads to curiosity and further conversation, we saw EHL knowledge leading directly to the development of skills and SE for environmental health in training participants. Like knowledge, relevant skills and SE are specific to the community context and local risks, and they should be defined and evaluated accordingly. There may be several specific types of SE to measure for a single environmental health risk community. For example, a study may want to know participants’ SE for sampling water and SE for talking to others about water quality results, to fully understand community members’ capability to address an environmental health risk.

Just as Cornell Lab of Ornithology has developed modifiable Likert scales for any specific SE type [45], which were used effectively in this study, generalizable evaluation tools for the knowledge-to-skills continuum of EHL should be further developed and tested. Finn and O’Fallon suggest a model that is based on Bloom’s taxonomy to assess literacy development for specific environmental health issues [10], which is already being applied in the field [17]. Their model suggests that actions taken at various levels of EHL could be an important indicator of EHL development.

#### 4.3.3. Community Change

If a project to increase EHL in an environmental health risk community does not ultimately aim to reduce harmful exposures, it is difficult to identify how the project might benefit the community. Although many EHL programs, including this one, have focused on individual- and household-level actions to reduce environmental exposures, reducing environmental health risks at their primary sources (industry, mining, city or state land use decisions, e.g.,) require larger coordinated efforts. Some environmental health researchers have used community-based approaches not only as a purposeful tool for research, but also for community-level action [69,70,71,72,73], and academics are well positioned to support environmental health risk communities in making systemic change. Explicitly defining community change as the highest level of EHL mandates that EHL interventions measure their success at least partially on whether positive community change transpires. This may mark a significant shift in programs to increase EHL moving forward, towards closer alignment with approaches such as community-based participatory research (CBPR), which state community action grounded in research findings as an explicit goal [74]. Although many studies have successfully used qualitative and community-based research methods to elucidate the more complex and dynamic factors influencing environmental health, including social factors, these methods remain underrepresented in the environmental health field [75,76].

Community action was not a focus of the trainings discussed here, as facilitator-scientists understood the sensitive nature of their role as outsiders and wanted to position themselves as supporters rather than instigators. However, these trainings were designed in part to recruit volunteers for an environmental health citizen science project, which was envisioned as the community action that training participants would be excited to pursue. Although hands-on practice with sampling methods was included in the training, and a significant increase in SE for doing science was observed post-training, it was surprising that only nine training participants signed up as citizen scientists. However, harvesting rainwater at home was a requirement to sign up as a citizen scientist, as the project involves monitoring harvested rainwater, and only twenty training participants indicated that they were currently harvesting rainwater at home. Thus, just under half of eligible participants (9 out of 20) did continue with the “action” part of the project. Over 160 residents of these four communities did ultimately volunteer and are currently acting as citizen scientists, however their recruitment to the project largely came through one of the designated project promotoras or other local leaders, rather than through attending a training. Acknowledging the barriers to citizen science participation that persist especially for diverse community members [77], successful recruitment of diverse participants for Project Harvest suggests the promotora model as a recommended method [43,78], which should be applied and studied further.

The hesitation expressed by a few training participants to learn about pollution on their property indicates a potential barrier to community-level environmental health action. Their concerns were primarily the potential decreased property values or legal responsibilities if unsafe contaminant levels were found. Additionally, especially in active mining communities, environmental monitoring can be perceived as a direct challenge to the status quo, potentially threatening jobs and economic viability in the area. Particularly in small communities, social pressure might inhibit a community members’ intrinsic motivation to act despite their concerns about health risk.

By creating a supportive environment for shared learning and relationship building, these trainings allowed community members and university researchers to gain understanding and trust, which sets the stage for further collaboration. Academics engaging directly with members of environmental risk communities in shared learning is in itself a community action, which allows alliances to be built and knowledge to be shared across cultural divides. We hope this study will motivate other environmental health researchers to invest in contextual learning with members of the communities who could most benefit from, and best inform, their research.

## 5. Conclusions

This study reveals a unique approach to both increasing EHL in environmental health risk communities and opening the door for community-academic partnerships for environmental health research and action. Although this in-person approach requires a considerable time commitment from both university researchers and community members, both parties benefit from the investment. Community members increase EHL specific to local risks, network with others interested in environmental health learning and action, build relationships with university researchers, and inform the direction of future research. University researchers share their professional knowledge directly with those for whom it is most personally relevant, build relationships with potential future research collaborators, and learn local knowledge and context to inform future study. In this study, awareness of cultural context (setting, language, cultural beliefs) was the most critical aspect of a successful training program to increase EHL. By meeting members of environmental health risk communities “where they’re at”, quite literally, academics position themselves to work alongside the public for greater impact of their research.

## Figures and Tables

**Table 1:** Partnering Communities Demographics, Environmental Health Risk, and Partnerships.

City/Town and County	Population ^1^	Median Household Income ^2^	Predominant Races/Ethnicities Represented ^1^	% Spanish-Speaking Households ^2^	Sources of Environmental Health Risk Recognized by Community ^5^	Prior/Current Partnerships
Tucson, Pima	520,116	$37,973	White, not Hispanic/Latino: 47.2% Hispanic/Latino: 41.6	28.8	Tucson International Airport Area Superfund Site [29], where aircraft and electronics manufacturing, fire drill training, and an unlined landfill have contributed trichloroethylene (TCE) and other contaminants observed in soil, groundwater, and municipal water [30,31]	Prior collaboration with non-profit organization Sonoran Environmental Research Institute (SERI), a community participatory research institute with extensive experience working with low-income Tucsonans around environmental health issues
Hayden ^3^, Gila	662	$36,094	Hispanic/Latino (of any race): 84.4%	61.8	ASARCO Hayden Plant Alternative Superfund Site, which includes the ASARCO smelter, concentrator, former Kennecott smelter and all associated tailings facilities [32] In 2016, ASARCO was involved in a $150 million settlement with the US Department of Justice and US Environmental Protection Agency for violations of the Clean Air Act [33]	National Institute of Environmental Health Sciences Superfund Research Program partnership School superintendent enthusiastic about gardening and rainwater harvesting served as local “champion” [34] to involve teachers and students in environmental health learning
Winkelman ^3^, Gila	353	$45,000	Hispanic/Latino (of any race): 84.2%	55.6		
Globe ^4^, Gila	7532	$42,557	White, not Hispanic/Latino: 55.3% Hispanic/Latino (of any race): 36.8%	14.9	Active copper smelter, rod mill, and open pit mine in Miami [35] The Mountain View Mobile Home Estates in Globe, AZ, sits on the site of a former chrysotile asbestos mill. This site was on the Superfund Program’s National Priorities List (NPL) due to asbestos contamination of soil and groundwater until clean-up activities were completed in 1988 [36]	Gila County Cooperative Extension agent became a local “champion” for environmental health learning [34], and successfully spread the enthusiasm to the Globe-Miami community
Miami ^4^, Gila	1837	$36,298	Hispanic/Latino (of any race): 56% White, not Hispanic/Latino: 40.6%	23.6		
Dewey-Humboldt, Yavapai	3894	$50,173	White, not Hispanic/Latino: 85.5%	5.2	Iron King Mine—Humboldt Smelter Superfund Site, which includes approximately four million cubic meters of mine tailings from legacy mine and smelter [37] 2012–2013 analyses of drinking water in local homes demonstrated arsenic above the US EPA drinking water standard (10 µg/L) [38]	Community members participated in past UA research projects: 1. Citizen science project Gardenroots related to soil contamination and backyard food gardens [11,39,40,41] 2. Biomonitoring project related to metal exposure in homes [42]

^1^ 2010 US Census; ^2^ 2012–2016 American Community Survey (ACS) 5 year estimate; ^3^ Although these neighboring municipalities joined as one community for this study, they are separated in Census data collection (Town of Hayden and Town of Winkelman); ^4^ Although these neighboring municipalities joined as one community for this study, they are separated in Census data collection (City of Globe and Town of Miami). ^5^ More information about sources of environmental health risk in these communities is available from the US EPA EJSCREEN tool, www.ejscreen.epa.gov/mapper/, and from the US EPA Toxic Release Inventory (TRI) Program database, https://www.epa.gov/toxics-release-inventory-tri-program.

**Table 2:** Training Participant Engagement Methods by Community.

Engagement Activity	Tucson	Hayden/Winkelman	Globe/Miami	Dewey-Humboldt
Press Releases		X	X	X
Local Newspaper		X	X	X
Town Newsletter				X
Cooperative Extension		X	X	X
Master Gardeners	X	X	X	X
Federal Superfund Site Meetings	X	X		X
Community Advisory Boards	X	X		X
School Superintendent & Teachers	X	X		X
SERI participants	X			
City of Tucson Water program participants	X			

**Table 3:** Research goals and associated data analysis methods.

**Research goal:** Gain understanding about training participants’ (1) initial motivation to learn about environmental health, and (2) attitude towards the environment.		
Assessment Category	Data	Survey Responses Coded for:
1. Motivation to learn	Four short-answer questions (pre-survey only)	Themes (qualitative)
2. Attitude towards the environment	Two multiple choice questions (pre- and post-survey)	Level of pro-environmental attitude (quantitative)
**Research goal:** Measure change in training participant EHL, as comprised of (3) environmental science knowledge, (4) skills and (5) motivation for environmental health action, (6) self-efficacy, and (7) community action for systemic change.		
Assessment Category	Data	Survey Responses Coded for:
3. Environmental science knowledge	Four multiple choice questions, one matching question, one rank order question, seven short answer questions (pre- and post-survey)	Level of understanding (quantitative), and for themes in specific knowledge concepts (qualitative)
4. Skills for environmental health	One multiple choice question, three short answer questions (pre- and post-survey)	Level of knowledge (quantitative), and for themes in specific knowledge concepts (qualitative)
5. Motivation for environmental action	Eleven Likert-scale items (pre- and post-survey)	Level of motivation (quantitative)
6. Self-efficacy (SE)	Six Likert-scale items measure SE for learning science, four items measure SE for doing science, twelve items measure SE for environmental action (pre- and post-survey)	Level of self-efficacy (quantitative)
7. Community change	Two short answer questions (pre- and post-survey) one short answer question (post-survey only) facilitator-scientist survey responses	Themes of political advocacy, teaching others, meeting/talking/networking with others, or other collective strategies. ^1^
**Research goal:** Gain understanding of (8) training participants’ experiences in the training, and (9) facilitator-scientists’ experiences in the training.		
Assessment Category	Data	Survey Responses Coded for:
8. Participant experience	Three short answer questions (post-survey only)	Themes (qualitative)
9. Facilitator-scientist experience	Open-ended survey conducted via email with a subset of the facilitator-scientists	Themes (qualitative)

^1^ Volunteering as a citizen scientist for Project Harvest post-training considered as a collective strategy.

**Table 4:** Level of knowledge coding rule and example responses to open-ended survey question.

Code	0—No Knowledge	1—Partial Knowledge	2—Baseline Knowledge	3—Advanced Knowledge
**Coding Rule**	Response is blank or reflects no knowledge of key concepts.	Response suggests some correct knowledge of topic but does not identify key concept.	Response describes key concept and is otherwise correct.	Response describes key concept with higher complexity or details.
Example question: How is the use of energy derived from coal (electricity) and climate change related?				
**Example Response**	“*Climate Change is the Glaciers melting, Didn’t understand!*”	“*Dirty air and chemicals from burning coal*”	“*Increase in CO_2_ green house gases*”	“*Burning of fossil fuels is the main contributor of rapidly increasing atmospheric CO_2_*”

**Table 5:** Level of environmental science knowledge pre- and post-training, all participants.

Survey Question # and Content	Mean Pre-Training Score ^1^	Mean Post-Training Score ^1^
1. Greenhouse gases	1.37	1.45
2–4. Impacts of climate change	1.79	2.42 *
6. Fossil fuel use impacts	1.00	1.74 *
8. Scientific method ^2^	0.84	0.87
9. Contaminant transport ^2^	0.92	0.97
10. Soil composition ^2^	0.95	0.97
14–16. Soil/water contamination	1.87	2.23
17a. Chemical concentrations	1.45	2.32 *
17b. Chemical concentrations	0.92	2.05 *

* Significant change pre-post (*p* < 0.01); ^1^ Responses were assigned one of the following: 0 = No Knowledge, 1 = Partial Knowledge, 2 = Baseline Knowledge, and 3 = Advanced Knowledge. ^2^ This was a right/wrong question, which could only be scored as 0 = Incorrect, 1 = Correct.

**Table 6:** Level of environmental science knowledge in “below-baseline pre” participants only.

Survey Question # and Content	Below- Baseline Participant *n*	Mean Pre-Training Score ^1^	Mean Post-Training Score ^1^
1. Greenhouse gases	20	0.80	1.10
2–4. Impacts of climate change	13	0.77	1.92 *
6. Fossil fuel use impacts	27	0.37	1.44 *
14–16. Soil/water contamination	13	0.31	1.85 *
17a. Chemical concentrations	21	0.24	2.10 *
17b. Chemical concentrations	32	0.22	1.88 *

* Significant change pre-post (*p* < 0.01); ^1^ Responses were assigned one of the following: 0 = No Knowledge, 1 = Partial Knowledge, 2 = Baseline Knowledge, and 3 = Advanced Knowledge.

**Table 7:** Wilcoxon Signed-Rank Test Results for Participants’ Pre-Post Self-Efficacy (SE) Change.

Self-Efficacy (SE) Type	Mean Pre	Mean Post
SE for learning science	2.70	3.76 *
SE for doing science	2.63	3.47 *
SE for environmental action	3.53	4.00 *

* Significant change pre-post (*p* < 0.01).

**Table 8:** Participant survey responses related to community action.

Type of Community Action	Examples
Political/Social Advocacy	“*Learn, listen, and organize with people & neighborhood*” —Tucson participant “f possible share emails to be able to get in touch with others” —Tucson participant “*Call political representatives to let them know we need to keep the EPA intact*”—Globe/Miami participant
Teach Others	“*Networking, collaboration and information to help educate others in our community including in our school garden program*” —Dewey-Humboldt participant “*So much info! Shared with grandparents who already harvest water*” —Hayden/Winkelman participant

## Supplementary Materials

The following are available online at http://www.mdpi.com/1660-4601/15/10/2203/s1, more information about Project Harvest can be found at projectharvest.arizona.edu. Instructional videos on water, soil, and plant sampling methods taught for home environmental contamination analysis can be found at https://projectharvest.arizona.edu/get-started#videos, S1: Recruitment flyers in English and Spanish, S2: Agendas from community trainings, S3: Pre-program survey, English version, S4: Content Survey, English version, S5: Post-program survey, English version.

## References

[1] National Environmental Justice Advisory Council (NEJAC) (2004). Ensuring Risk Reduction in Communities with Multiple Stressors: Environmental Justice and Cumulative Risks/Impacts.

[2] Bullard R.D. (2008). Dumping in Dixie: Race, Class, and Environmental Quality.

[3] Gee G.C., Payne-Sturges D.C. (2004). Environmental Health Disparities: A Framework Integrating Psychosocial and Environmental Concepts. Environ. Health Perspect..

[4] Frumkin H., Hess J., Luber G., Malilay J., McGeehin M. (2008). Climate Change: The Public Health Response. Am. J. Public Health.

[5] Wilson S.M., Richard R., Joseph L., Williams E. (2010). Climate Change, Environmental Justice, and Vulnerability: An Exploratory Spatial Analysis. Environ. Justice.

[6] Oleson K.W., Monaghan A., Wilhelmi O., Barlage M., Brunsell N., Feddema J., Hu L., Steinhoff D.F. (2015). Interactions between urbanization, heat stress, and climate change. Clim. Chang..

[7] Bullard R.D. (2001). Environmental Justice in the 21st Century: Race Still Matters. Phylon.

[8] Gray K.M. (2018). From Content Knowledge to Community Change: A Review of Representations of Environmental Health Literacy. Int. J. Environ. Res. Public Health.

[9] Hoover A. Connecting Disciplines to Inform and Develop the Emerging Field of Environmental Health Literacy. https://www.niehs.nih.gov/research/supported/assets/docs/a_c/connecting_disciplines_to_inform_and_develop_the_emerging_field_of_environmental_health_literacy_508.pdf.

[10] Finn S., O’Fallon L. (2017). The Emergence of Environmental Health Literacy—From Its Roots to Its Future Potential. Environ. Health Perspect..

[11] Ramirez-Andreotta M., Brody J., Lothrop N., Loh M., Beamer P., Brown P. (2016). Reporting back environmental exposure data and free choice learning. Environ. Health.

[12] Zarcadoolas C., Timm E., Bibeault L. (2001). Brownfields: A case study in partnering with residents to develop an easy-to-read print guide. J. Environ. Health.

[13] Hoover A.G., Finn S., O’Fallon L.R. (2019). Defining Environmental Health Literacy. Environmental Health Literacy.

[14] Society for Public Health Education What Is Environmental Health Literacy?. http://www.sophe.org/environmentalhealth/key_ehl.asp.

[15] White B.M., Hall E.S., Johnson C. (2014). Environmental Health Literacy in Support of Social Action: An Environmental Justice Perspective. J. Environ. Health.

[16] Stokes S.C., Hood D.B., Zokovitch J., Close F.T. (2010). Blueprint for Communicating Risk and Preventing Environmental Injustice. J. Health Care Poor Underserv..

[17] Madrigal D.S., Minkler M., Parra K.L., Mundo C., Gonzalez J.E.C., Jimenez R., Vera C., Harley K.G. (2016). Improving Latino Youths’ Environmental Health Literacy and Leadership Skills through Participatory Research on Chemical Exposures in Cosmetics: The HERMOSA Study. Int. Q. Community Health Educ..

[18] Corburn J. (2007). Community knowledge in environmental health science: Co-producing policy expertise. Environ. Sci. Policy.

[19] Falk J. (2002). The Contribution of Free-Choice Learning to Public Understanding of Science. Interciencia.

[20] Falk J.H., Dierking L.D. (2000). Learning from Museums: Visitor Experiences and the Making of Meaning.

[21] Falk J., Storksdieck M. (2007). Using the Contextual Model of Learning to Understand Visitor Learning from a Science Center Exhibition. Sci. Educ..

[22] Colucci-Gray L., Perazzone A., Dodman M., Camino E. (2013). Science education for sustainability, epistemological reflections and educational practices: From natural sciences to trans-disciplinarity. Cult. Stud. Sci. Educ..

[23] Lee J.E.C., Lemyre L., Mercier P., Bouchard L., Krewski D. (2005). Beyond the Hazard: The Role of Beliefs in Health Risk Perception. Hum. Ecol. Risk Assess..

[24] Johnson E.B. (2002). Contextual Teaching and Learning: What It Is and Why It’s Here to Stay.

[25] Zemits B., Maypilama L., Wild K., Mitchell A., Rumbold A. (2015). Moving Beyond “Health Education”: Participatory Filmmaking for Cross-Cultural Health Communication. Health Commun..

[26] Ratnapradipa D., Middleton W.K., Wodika A.B., Brown S.L., Preihs K. (2015). What Does the Public Know About Environmental Health? A Qualitative Approach to Refining an Environmental Health Awareness Instrument. J. Environ. Health.

[27] Adams C., Brown P., Morello-Frosch R., Brody J.G., Rudel R., Zota A., Dunagan S., Tovar J., Patton A.S. (2011). Disentangling the Exposure Experience: The Roles of Community Context and Report-Back of Environmental Exposure Data. J. Health Soc. Behav..

[28] Shirk J.L., Ballard H.L., Wilderman C.C., Phillips T., Wiggins A., Jordan R., McCallie E., Minarchek M., Lewenstein B.V., Krasny M.E. (2012). Public Participation in Scientific Research: A Framework for Deliberate Design. Ecol. Soc..

[29] US Environmental Protection Agency Tucson International Airport Area National Priorities List and Superfund Alternative Approach Sites. https://cumulis.epa.gov/supercpad/cursites/csitinfo.cfm?id=0900684.

[30] Rodenbeck S.E., Maslia M.L. (1998). Groundwater Modeling and GIS to Determine Exposure to TCE at Tucson. Pract. Period. Hazard. Toxic Radioact. Waste Manag..

[31] Conforma Tech, Inc (2014). Phase 1 Environmental Site Assessment: Los Reales Landfill Expansion Project. https://www.tucsonaz.gov/files/es/P-I_ESA_Los_Reales_Expansion_Project.pdf.

[32] US Environmental Protection Agency ASARCO Hayden Plant National Priorities List and Superfund Alternative Approach Sites. https://cumulis.epa.gov/supercpad/cursites/csitinfo.cfm?id=0900497.

[33] (1970). Clean Air Act. https://www.epa.gov/clean-air-act-overview/clean-air-act-text.

[34] Gallagher D.R. (2009). Advocates for environmental justice: The role of the champion in public participation implementation. Local Environ..

[35] Freeport McMoRan Miami AZ Operations. http://www.freeportinarizona.com/our-company/az-operations/miami/.

[36] US Environmental Protection Agency Mountain View Mobile Home Estates National Priorities List and Superfund Alternative Approach Sites. https://cumulis.epa.gov/supercpad/cursites/csitinfo.cfm?id=0900679.

[37] US Environmental Protection Agency Iron King Mine—Humboldt Smelter National Priorities List and Superfund Alternative Approach Sites. https://cumulis.epa.gov/supercpad/cursites/csitinfo.cfm?id=0905049.

[38] Loh M.M., Sugeng A., Lothrop N., Klimecki W., Cox M., Wilkinson S.T., Lu Z., Beamer P.I. (2016). Multimedia exposures to arsenic and lead for children near an inactive mine tailings and smelter site. Environ. Res..

[39] Ramirez-Andreotta M.D., Lothrop N., Wilkinson S.T., Root R.A., Artiola J.F., Klimecki W., Loh M. (2016). Analyzing patterns of community interest at a legacy mining waste site to assess and inform environmental health literacy efforts. J. Environ. Stud. Sci..

[40] Ramirez-Andreotta M.D., Brusseau M.L., Beamer P., Maier R.M. (2013). Home gardening near a mining site in an arsenic-endemic region of Arizona: Assessing arsenic exposure dose and risk via ingestion of home garden vegetables, soils, and water. Sci. Total Environ..

[41] Sandhaus S. Evaluating the Motivations, Knowledge, and Efficacy of Participants in Environmental Health Citizen Science Projects. https://repository.arizona.edu/handle/10150/625311.

[42] Ramirez-Andreotta M.D., Brody J.G., Lothrop N., Loh M., Beamer P.I., Brown P. (2016). Improving Environmental Health Literacy and Justice through Environmental Exposure Results Communication. Int. J. Environ. Res. Public Health.

[43] Sandhaus S., Ramírez-Andreotta M.D., Kilungo A., Wolf A.M., Sandoval F., Henriquez P. (2018). Combating Climate Injustices: An Informal Science and Popular Education Approach to Addressing Environmental Health Disparities, Combating Climate Injustices: An Informal Science and Popular Education Approach to Addressing Environmental Health Disparities. Pedagogy Health Promot..

[44] IBM Corp (2017). IBM SPSS Statistics for Windows 25.

[45] Phillips T., Minarchek M., Porticella N., Shirk J., Wilderman C., Ellenbogen K., Bonney R. (2014). DEVISE: Building Evaluation Capacity in PPSR and Informal Science Education.

[46] Bandura A. (1982). Self-efficacy mechanism in human agency. Am. Psychol..

[47] U.S. Department of Health and Human Services, Agency for Toxic Substances and Disease Registry Health Consultation: Dioxin Contaminated Aerial Spraying Landing Locations Kellner, Icehouse, and Sixshooter Canyons. https://www.fs.usda.gov/Internet/FSE_DOCUMENTS/stelprdb5328294.pdf.

[48] Shoecraft B. (1971). Sue the Bastards!.

[49] Tourangeau R., Yan T. (2007). Sensitive questions in surveys. Psychol. Bul..

[50] Urdan T., Pajares F. (2006). Self-Efficacy Beliefs of Adolescents.

[51] Deci E.L., Ryan R.M. (2008). Self-determination theory: A macrotheory of human motivation, development, and health. Can. Psychol..

[52] (2017). Cornell Lab of Ornithology Evaluation Research Technical Brief: Motivation for Environmental Action (Custom). https://cornell.qualtrics.com/jfe/form/SV_cGxLGl1AlyAD8FL?Q_DL=byCDh4YfnimQ1AV_cGxLGl1AlyAD8FL_MLRP_6mctE0qCqCRktjT&Q_CHL=email.

[53] Bingle W.H., Gaskell P.J. (1994). Scientific literacy for decision making and the social construction of scientific knowledge. Sci. Educ..

[54] Brown B.A., Reveles J.M., Kelly G.J. (2005). Scientific literacy and discursive identity: A theoretical framework for understanding science learning. Sci. Educ..

[55] Sadler T.D. (2009). Situated learning in science education: Socio-scientific issues as contexts for practice. Stud. Sci. Educ..

[56] Bramston P., Pretty G., Zammit C. (2011). Assessing Environmental Stewardship Motivation. Environ. Behav..

[57] Neuhauser L. (2017). Integrating participatory design and health literacy to improve research and interventions. Inform. Serv. Use.

[58] Pezzullo P.C., Cox R. (2017). Environmental Communication and the Public Sphere.

[59] Colucci-Gray L. (2014). Beyond evidence: A critical appraisal of global warming as a socio-scientific issue and a reflection on the changing nature of scientific literacy in school. Cult. Stud. Sci. Educ..

[60] Carlone H.B., Huffling L.D., Tomasek T., Hegedus T.A., Matthews C.E., Allen M.H., Ash M.C. (2015). ‘Unthinkable’ Selves: Identity boundary work in a summer field ecology enrichment program for diverse youth. Int. J. Sci. Educ..

[61] Allen B.L. (2004). Shifting boundary work: Issues and tensions in environmental health science in the case of Grand Bois, Louisiana. Sci. Cult..

[62] Harvey H.D., Fleming P. (2003). The readability and audience acceptance of printed health promotion materials used by environmental health departments. J. Environ. Health.

[63] Fitzpatrick-Lewis D., Yost J., Ciliska D., Krishnaratne S. (2010). Communication about environmental health risks: A systematic review. Environ. Health.

[64] Wenger E. (2011). Communities of Practice: A Brief Introduction.

[65] González-Howard M., McNeill K.L. (2016). Learning in a community of practice: Factors impacting English-learning students’ engagement in scientific argumentation. J. Res. Sci. Teach.

[66] Olitsky S. (2007). Promoting student engagement in science: Interaction rituals and the pursuit of a community of practice. J. Res. Sci. Teach..

[67] Allen B.L. (1998). Women Scientists and Feminist Methodologies in Louisiana’s Chemical Corridor.

[68] DeLemos J., Rock T., Brugge D., Slagowski N., Manning T., Lewis J. (2007). Lessons from the Navajo: Assistance with Environmental Data Collection Ensures Cultural Humility and Data Relevance. Prog. Community Health Partnersh.

[69] Ali R., Olden K., Xu S. (2008). Community-Based Participatory Research: A Vehicle to Promote Public Engagement for Environmental Health in China. Environ. Health Perspect..

[70] Ablah E., Brown J., Carroll B., Bronleewe T. (2016). A Community-Based Participatory Research Approach to Identifying Environmental Concerns. J. Environ. Health.

[71] Gonzalez P.A., Minkler M., Garcia A.P., Gordon M., Garzón C., Palaniappan M., Prakash S., Beveridge B. (2011). Community-based participatory research and policy advocacy to reduce diesel exposure in West Oakland, California. Am. J. Public Health.

[72] Commodore A., Wilson S., Muhammad O., Svendsen E., Pearce J. (2017). Community-based participatory research for the study of air pollution: A review of motivations, approaches, and outcomes. Environ. Monit. Assess..

[73] Ponder-Brookins P., Witt J., Steward J., Greenwell D., Chew G.L., Samuel Y., Kennedy C., Brown M.J. (2014). Incorporating Community-Based Participatory Research Principles into Environmental Health Research: Challenges and Lessons Learned from a Housing Pilot Study. J. Environ. Health.

[74] Ramirez-Andreotta M.D., Brusseau M.L., Artiola J.F., Maier R.M., Gandolfi A.J. (2014). Environmental Research Translation: Enhancing interactions with communities at contaminated sites. Sci. Total Environ..

[75] Scammell M.K. (2010). Qualitative Environmental Health Research: An Analysis of the Literature, 1991–2008. Environ. Health Perspect..

[76] Baron S., Sinclair R., Payne-Sturges D., Phelps J., Zenick H., Collman G.W., O’Fallon L.R. (2009). Partnerships for Environmental and Occupational Justice: Contributions to Research, Capacity and Public Health. Am. J. Public Health.

[77] Pandya R.E. (2012). A framework for engaging diverse communities in citizen science in the US. Front. Ecol. Environ..

[78] Ramirez-Andreotta M., Finn S., O’Fallon L.R. (2019). Engaging with Ethnically Diverse Community Groups. Environmental Health Literacy.

