# Supplementary material for: Increasing Environmental Health Literacy through Contextual Learning in Communities at Risk
Source: Int J Environ Res Public Health. 2018 Oct 9;15(10):2203. doi: 10.3390/ijerph15102203 (PMC6210322; doi:10.3390/ijerph15102203)
Supplement: Supplementary file 1 [file ijerph-15-02203-s001.zip › S1_RecruitmentDeweyHumboldtEnglish.pdf]

Project Harvest

WANT TO LEARN MORE ABOUT YOUR HARVESTED WATER, SOIL, & PLANT DATA?

MARK YOUR CALENDARS FOR STEP 1 OF PROJECT HARVEST!

Dewey - Humboldt, AZ Training

August 12-13, 2017
8:00 - 5:00PM

Humboldt Elementary School, Room #7
2750 Corral St, Humboldt, AZ 86329

Step 1: Learn about climate change, health, and environmental quality. Learn how to install a water harvesting system, and be trained in how to collect harvested water, soil, and/or vegetable samples from your garden for environmental analysis. Meet others in your community who are interested in environmental and food quality.

For more information, please contact:
Mónica Ramírez-Andreotta, PhD
mdramire@email.arizona.edu
520-621-0091

COLLEGE OF AGRICULTURE & LIFE SCIENCES
Soil, Water and Environmental Science

Sonora Environmental Research Institute, Inc.

This material is based upon work supported by the National Science Foundation under Grant No. DRL-1612554. Any opinions, findings, and conclusions or recommendations expressed in this material are those of the author(s) and do not necessarily reflect the views of the National Science Foundation.

Visit our website to register:
http://projectharvest.arizona.edu/
