# Supplementary material for: Increasing Environmental Health Literacy through Contextual Learning in Communities at Risk
Source: Int J Environ Res Public Health. 2018 Oct 9;15(10):2203. doi: 10.3390/ijerph15102203 (PMC6210322; doi:10.3390/ijerph15102203)
Supplement: Supplementary file 1 [file ijerph-15-02203-s001.zip › S1_RecruitmentTucsonSpanish.pdf]

Project Harvest

¿QUIERE SABER M S SOBRE LOS DATOS DEL AGUA QUE RECOLECTA, EL SUELO, Y LAS PLANTAS?

MARQUE SU CALENDARIO PARA TOMAR EL PRIMER PASO DE "PROJECT HARVEST"

Entrenamiento en Tucson, AZ

22-26 de Mayo del 2017
8:30 - 12:30 PM

Tucson City Council Office Ward 6,
3202 E 1st St, Tucson, AZ 85716

Step 1: Aprenda como instalar un sistema de recolección de agua de lluvia y reciba entrenamiento de como tomar muestras de agua recolectada, suelo y/o vegetales de sus jardines para análisis ambientales (análisis microbianos, metales y compuestos orgánicos). Conozca a otros en su comunidad que estén interesados en la calidad del medio ambiente y de los alimentos.

Para obtener más información, favor de ponerse en contacto con:

Mónica Ramírez-Andreotta, PhD
mdramire@email.arizona.edu
520-621-0091

Visita nuestro sitio web para registrarse:
http://projectharvest.arizona.edu/

THE UNIVERSITY OF ARIZONA
College of Agriculture & Life Sciences
Soil, Water & Environmental Science

Sonora Environmental Research Institute, Inc.

This material is based upon work supported by the National Science Foundation under Grant No. DRL-1612554. Any opinions, findings, and conclusions or recommendations expressed in this material are those of the author(s) and do not necessarily reflect the views of the National Science Foundation.
