# Supplementary material for: Increasing Environmental Health Literacy through Contextual Learning in Communities at Risk
Source: Int J Environ Res Public Health. 2018 Oct 9;15(10):2203. doi: 10.3390/ijerph15102203 (PMC6210322; doi:10.3390/ijerph15102203)
Supplement: Supplementary file 1 [file ijerph-15-02203-s001.zip › S2_Training Agendas.pdf]

Appendix B

Tucson 5-day Training Agenda

	**May 22 - Monday**	**May 23 - Tuesday**	**May 24 - Wednesday**	**May 25 - Thursday**	**May 26 - Friday**
**8:30 – 8:40**	**Welcome!**				
**8:40-9:00**	**Pre-Survey**	**Questions and Reflections**			
**9:00 - 10:30**	**Introduction to Project Harvest and Climate Change** - Monica Ramirez-Andreotta	**Energy: Consumption, Conservation and Efficiency** - Monica Ramirez-Andreotta	**Higher Temperatures, Extreme Heat Events, and Effects on Air Quality** - Eduardo Saez	**Microbiology: Water, Soil, and Food Quality** - Aminata Kilungo & Jean McLain	**Rainwater Harvesting & Sampling Protocols at Nottinghill Community Garden,** 2660 N Alvernon Way, Tucson, AZ 85712
**10:30-10:40**	BREAK	BREAK	BREAK	BREAK	
**10:40 - 12:20**	**Introduction to Rainwater Harvesting** - Flor Morales	**Chemistry: Organic Compounds and Water Quality** - Leif Abrell	**Chemistry: Inorganic pollutants, Water, soil, and Plant Quality** - Monica Ramirez-Andreotta & Rob Root	**Introduction to Sampling Protocols**	
**12:20-12:30**	Summary of the Day	Summary of the Day	Summary of the Day	Summary of the Day	Closing & Post-Survey

Globe-Miami 5-day Training Agenda

	June 12 Monday	June 13 Tuesday	June 14 Wednesday	June 15 Thursday	June 16 Friday
**8:30 – 8:40**	**Welcome!**				
**8:40-9:00**	**Pre-Survey**	**Questions and Reflections**			
**9:00 - 10:30**	**Introduction to Project Harvest and Climate Change** - Monica Ramirez-Andreotta	**Higher Temperatures, Extreme Heat Events, and Effects on Air Quality** - Eduardo Saez	**Microbiology: Water, Soil, and Food Quality** - Aminata Kilungo and Jean McLain	**Energy: Consumption, Conservation and Efficiency** - Janick Artiola	**Rainwater Harvesting & Sampling Protocols at Bullion Plaza**

**10:30-10:40**	BREAK	BREAK	BREAK	BREAK	**Cultural Center & Museum,** 150 N Plaza Circle, Miami, AZ 85539
**10:40 - 12:20**	**Introduction to Rainwater Harvesting** - Flor Morales	**Chemistry: Inorganic pollutants, Water, soil, and plant quality** - Monica Ramirez-Andreotta	**Chemistry: Organic Compounds and Water Quality** - Leif Abrell	**Introduction to Sampling Protocols**	
**12:20-12:30**	Summary of the Day	Summary of the Day	Summary of the Day	Summary of the Day	Closing & Post-Survey

Hayden-Winkleman 3-day Training Agenda

**July 25 - Tuesday**	
8:30 - 8:40	**Welcome**
8:45 - 9:30	Pre-Survey
9:30 - 11:00	**Introduction to Project Harvest and Climate Change** - Monica Ramirez
11:00 - 11:10	*Break*
11:10 - 12:30	**Introduction to Rainwater Harvesting -** Flor Sandoval
12:30 - 1:00	*Lunch*
1:00 - 2:15	**Chemistry: Inorganic Pollutants, and Water, Soil, and Plant Quality** - Monica Ramirez & Rob Root
2:15 - 2:50	
2:50 - 3:00	Summary of the Day

**July 26 - Wednesday**	
8:30 - 8:40	**Questions and Reflections**
8:45 – 10:30	**Effects of Global Climate Change: Temperature, Weather, and Air Pollution -** Eduardo Saez
10:30 – 10:45	*Break*
10:45 -12:30	**Microbiology: Water, Soil, and Food Quality** - Jean McLain
12:30 - 1:00	*Lunch*
1:00 - 2:50	**Sampling Protocols: Inorganic and Microbial contaminants**
2:50 - 3:00	Summary of the Day

July 27 - Thursday

8:30 - 8:40	**Questions and Reflections**
8:45 – 10:30	**Climate Change and Energy: Consumption, Conservation and Efficiency –** Janick Artiola
10:30 – 10:45	*Break*
10:45 -12:30	**Chemistry: Organic Compounds and Water Quality** - Leif Abrell
12:30 - 1:00	*Lunch*
1:00 - 2:00	**Sampling Protocols: Organic Compounds**
2:00 - 3:00	Post- Survey

Dewey-Humboldt 2-day Training Agenda

August 12 - Saturday	
**8:00 - 8:10**	**Welcome!**
**8:10 - 9:00**	Pre-Survey
**9:00 - 10:30**	**Introduction to Project Harvest and Climate Change** - Monica Ramirez
**10:30 - 10:40**	*Break*
**10:40 - 12:00**	**Introduction to Rainwater Harvesting** - Flor Sandoval
**12:00 - 12:30**	*Lunch*
**12:30 - 2:00**	**Effects of Global Climate Change: Temperature, Weather, and Air Pollution** - Eduardo Saez
**2:00-4:00**	**Chemistry: Inorganic Pollutants, and Water, Soil, and Plant Quality** - Monica Ramirez & Rob Root
**4:00 - 4:50**	**Sampling Protocols: Inorganic**
**4:50 - 5:00**	*Summary of the Day*
August 13 - Sunday	
**8:00 - 8:15**	Questions and Reflections
**8:15 - 9:45**	**Chemistry: Organic Compounds and Water Quality** - Leif Abrell
**9:45-10:00**	*Break*
**10:00 - 11:00**	**Sampling Protocol: Organic**
**11:00 - 12:30**	**Climate Change and Energy: Consumption, Conservation and Efficiency** - Janick Artiola
**12:30 - 1:00**	*Lunch*
**1:00 - 2:30**	**Microbiology: Water, Soil, and Food Quality** - Aminata Kilungo

**2:30 - 4:00**	**Sampling Protocol: Microbial**
**4:00 - 5:00**	Post-Survey
