# Supplementary material for: Increasing Environmental Health Literacy through Contextual Learning in Communities at Risk
Source: Int J Environ Res Public Health. 2018 Oct 9;15(10):2203. doi: 10.3390/ijerph15102203 (PMC6210322; doi:10.3390/ijerph15102203)
Supplement: Supplementary file 1 [file ijerph-15-02203-s001.zip › S3_PreProgramSurvey.pdf]

A little more about you……. **Pre-Program Survey**

1. Please tell us why you are participating in this program:

2. What do you hope to get out your participation in this program?

3. **Are you currently doing rainwater harvesting? (circle one)** YES NO

3a. **If not, is rainwater harvesting something you'll like to do? (circle one)** YES NO

3b. **If applicable, what are some of the obstacles keeping you from not doing it?**

4. **Do you now, or have you in the past, participated in any programs to learn more about the environment, water, or energy?** YES NO

If yes, please list the programs:

5. Describe what you can do outside and inside your home to protect the environment, conserve water, conserve energy, and protect the health of your family and neighbors.
