# Supplementary material for: Increasing Environmental Health Literacy through Contextual Learning in Communities at Risk
Source: Int J Environ Res Public Health. 2018 Oct 9;15(10):2203. doi: 10.3390/ijerph15102203 (PMC6210322; doi:10.3390/ijerph15102203)
Supplement: Supplementary file 1 [file ijerph-15-02203-s001.zip › S5_PostProgramSurvey.pdf]

ID ___________________________________ Date: ________________
(to be used by the external evaluator only for pairing surveys)

Project Harvest – Post Program Survey

Thank you for helping us evaluate this program. We are collecting information to better understand you, the participants, and any changes that develop in your knowledge and attitudes during this program. This data is being collected by an external evaluator, Dr. Sanlyn Buxner, from the College of Education at the University of Arizona.

1. What did you gain this week?

2. Do you plan to do rainwater harvesting? (circle one) YES NO Already doing it

2a. If applicable, what are some of the obstacles keeping you from not doing it?

2b. If applicable, what support do you need to be able to start rainwater harvesting?

3) What suggestions do you have for us to improve this workshop for the future?

4) What additional training would you like to further your own knowledge and skills related to this project?

5) Describe what you can do outside and inside your home to protect the environment, conserve water, conserve energy, and protect the health of your family and neighbors.

Inside	Outside

Name:__________________________

1. **Which gases contribute the most to climate change? (circle all the best responses)**
   CO_2 (carbon dioxide), H_2O (water), N_2 (nitrogen), O_2 (oxygen), Ar (argon), CH_4 (methane).

2. **What, if any, evidence indicates that climate change is occurring?**

3. **What, if any, evidence indicates that weather is changing?**

4. **What are major impacts of climate change on the Earth?**

5. **What are three things you can do to curb the effects of climate change?**

6. **How are the use of energy derived from coal (electricity), and climate change - related?**

7. **How can you save energy at home? (circle <u>all</u> the correct answers)**
   a. Changing an old refrigerator for a new one
   b. Planting a lawn in the yard
   c. Remove trees out of the yard
   d. Turning off lights and televisions when not in use
   e. Changing incandescent light bulbs for LED bulbs
   f. Increasing the temperature of the house in winter and lowering it in summer
   g. Lowering the temperature of the water heater
   h. Ensure that all windows and doors are sealed.

8. **As part of a laboratory experiment, five students measured the weight of the same leaf four times. They recorded 20 slightly different weights. All of the work was done carefully and correctly. Their goal was to be as accurate as possible and reduce error in the experiment to a minimum. Which of the following is the BEST method to report the weight of the leaf?**

   a. Ask the teacher to weigh the leaf.
   b. Report the first measurement.
   c. Average all of the weights that were recorded.
   d. Average the highest and lowest weights recorded.
   e. Discard the lowest five weights.

9. **Imagine a ship has wrecked at sea spilling toxic chemicals into the ocean. Could some of those chemicals end up in your body?**

   a. Yes
   b. No
   c. Not Sure

10. **Soils consist of:**
   a. Minerals
   b. Minerals, water, and air
   c. Minerals, microorganisms, and plant residues
   d. Minerals and plant residues
   e. Minerals, water, organic matter, air and microorganisms

11. **Below are some specific environmental proposals. For each one, please say whether you generally favor or oppose it.**

Setting more strict emissions and pollution standards for business and industry.	Favor	Oppose
More strongly enforcing federal environmental regulations.	Favor	Oppose
Spending more money on developing solar and wind power.	Favor	Oppose

12. **With which one of these statements do you most agree (please circle your answer):**

Protection of the environment should be given priority, even at the risk of curbing economic growth

OR

Economic growth should be given priority, even if the environment suffers to some extent

13. What changes in your life style can you make to produce a positive impact on water reliability for the future?

Related to this project

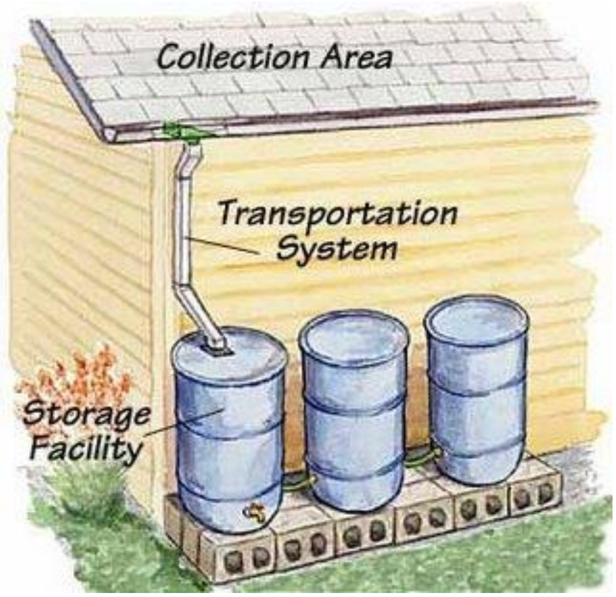

1. Why do we want to collect water and soil samples?

2. How can collected water and/or soils be contaminated?

3. How can your home location affect water and soil quality?

4. To the best of your ability, redraw each of the labeled circles below along the line to show which ones are bigger and smaller concentrations. You can draw above or below the line

smallest concentration ⟷ biggest concentration

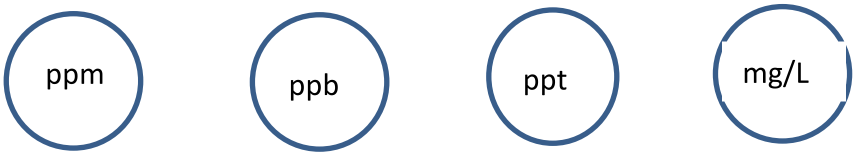

4b. Please write what you know about the abbreviations above:

4c. On the left is a measurement abbreviation and on the right is a definition. Match up the definition to the abbreviation.

	ppm	A. micrograms per liter
	ppb	B. nanograms per liter
	ppt	C. milligrams per liter

Name_____________________________________

Please rate how much you disagree or agree with each of the following statements by circling the number in the appropriate column

	**Strongly disagree**	**Disagree**	**Neutral**	**Agree**	**Strongly agree**
I think I'm pretty good at understanding topics about **water quality**.	1	2	3	4	5
I think I'm pretty good at understanding topics about **soil quality**.	1	2	3	4	5
I think I'm pretty good at understanding topics about **environmental science and climate change.**	1	2	3	4	5
I feel confident in my ability to explain **water quality** topics to others.	1	2	3	4	5
I feel confident in my ability to explain **climate change** to others.	1	2	3	4	5
I feel confident in my ability to explain **soil quality** topics to others.	1	2	3	4	5
I feel confident about my ability to explain how to **monitor water quality** to others.	1	2	3	4	5
I feel confident about my ability to explain how to **monitor soil quality** to others.	1	2	3	4	5
It takes me a long time to understand how to **monitor water quality**.	1	2	3	4	5
It takes me a long time to understand how to **monitor soil quality**.	1	2	3	4	5
I feel confident in my ability to help **protect water quality.**	1	2	3	4	5
I feel confident in my ability to help **protect soil quality.**	1	2	3	4	5
I feel confident in my ability to help address climate change.	1	2	3	4	5
I am capable of making a positive impact on **water quality.**	1	2	3	4	5
I am capable of making a positive impact on **soil quality.**	1	2	3	4	5
I am able to help take care of **water quality**.	1	2	3	4	5
I believe I can contribute to solutions to **water quality** problems by my actions.	1	2	3	4	5
I believe I can contribute to solutions to **climate change** problems by my actions.	1	2	3	4	5
I don't think I can make any different in solving **water quality** problems.	1	2	3	4	5
I don't think I can make any different in solving **soil quality** problems.	1	2	3	4	5
I don't think I can make any different in solving **climate change** problems.	1	2	3	4	5
I believe that I personally, working with others, can help **solve water issues**.	1	2	3	4	5

Think about some of the things you do to protect water quality or solve water quality problems. Why do you do these things?	**Strongly disagree**	**Disagree**	**Neutral**	**Agree**	**Strongly agree**
Because I think it’s a good idea to do something to protect **water quality.**	1	2	3	4	5
Because other people will be disappointed in me if I don't.	1	2	3	4	5
Because I'm concerned about what could happen to people I care about if I don't do anything.	1	2	3	4	5
Because I would feel guilty if I didn't do anything to protect **water quality.**	1	2	3	4	5
Because I enjoy doing it.	1	2	3	4	5
Because people I look up to think it's a really good thing to do.	1	2	3	4	5
Because I think it’s a good idea to protect **water quality.**	1	2	3	4	5
Because it's fun to do it.	1	2	3	4	5
For the recognition I get from others.	1	2	3	4	5
Because I think it’s important to take care of **water quality.**	1	2	3	4	5
Because I think it’s important to take care of **soil quality.**	1	2	3	4	5

Thank you for your time!
